# Corn leaf disease: insightful diagnosis using VGG16 empowered by explainable AI

**DOI:** 10.3389/fpls.2024.1402835

**Published:** 2024-06-26

**Authors:** Maria Tariq, Usman Ali, Sagheer Abbas, Shahzad Hassan, Rizwan Ali Naqvi, Muhammad Adnan Khan, Daesik Jeong

**Affiliations:** ^1^ Department of Computer Science, National College of Business Administration and Economics, Lahore, Pakistan; ^2^ Department of Computer Science, Lahore Garrison University, Lahore, Pakistan; ^3^ Department of Computer Science and Engineering, Sejong University, Seoul, Republic of Korea; ^4^ College of Computer Engineering and Science, Prince Mohammad Bin Fahd University, Al Khobar, Saudi Arabia; ^5^ Marine Engineering Department, Military Technological College, Muscat, Oman; ^6^ Department of Artificial Intelligence and Robotics, Sejong University, Seoul, Republic of Korea; ^7^ Department of Software, Faculty of Artificial Intelligence and Software, Gachon University, Seongnam, Republic of Korea; ^8^ College of Convergence Engineering, Sangmyung University, Seoul, Republic of Korea

**Keywords:** intelligent agriculture system, machine learning (ML), corn leaf disease, explainable artificial intelligence (XAI), Visual Geometry Group 16 (VGG16), layer-wise relevance propagation (LRP)

## Abstract

The agricultural sector is pivotal to food security and economic stability worldwide. Corn holds particular significance in the global food industry, especially in developing countries where agriculture is a cornerstone of the economy. However, corn crops are vulnerable to various diseases that can significantly reduce yields. Early detection and precise classification of these diseases are crucial to prevent damage and ensure high crop productivity. This study leverages the VGG16 deep learning (DL) model to classify corn leaves into four categories: healthy, blight, gray spot, and common rust. Despite the efficacy of DL models, they often face challenges related to the explainability of their decision-making processes. To address this, Layer-wise Relevance Propagation (LRP) is employed to enhance the model's transparency by generating intuitive and human-readable heat maps of input images. The proposed VGG16 model, augmented with LRP, outperformed previous state-of-the-art models in classifying corn leaf diseases. Simulation results demonstrated that the model not only achieved high accuracy but also provided interpretable results, highlighting critical regions in the images used for classification. By generating human-readable explanations, this approach ensures greater transparency and reliability in model performance, aiding farmers in improving their crop yields.

## 1 Introduction

**Figure 1:**
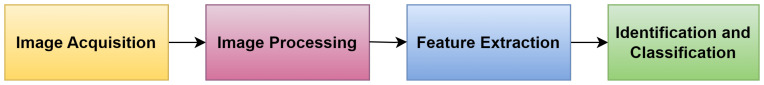
Steps involved in an intelligent agriculture system.

Economic development remains highly dependent on the agricultural sector (Mgomezulu et al., 2024), particularly in low-income nations where the industry significantly depends on the total labor force for income (Dethier and Effenberger, 2012). After rice and wheat, corn is one of the most important food crops in the world. People in central and south America generally get their carbohydrates from it. In the United States, corn is an essential alternate food source. Corn is a staple grain consumed by people in several Indonesian areas. In addition to providing humans with energy, corn is grown for animal feed, cooking oil is made from grains, and flour (sometimes known as cornstarch) is also made from grains). Corn and cob flour are also industrial raw materials (Kusumo et al., 2018). Corn is susceptible to many diseases, some of which can make it difficult for the crops to grow to their full potential (Widmer et al., 2024). The intensity of attacks on corn plants dictates how much of an impact it has. The disease usually causes irregular cell and tissue activity and stunted growth in affected plants. Some plants experience stunting and withering, while others show chromatic changes such as leaf drying or yellowing (Khoirunnisak, 2020). Early diagnosis mainly prevents and controls plant diseases, so agricultural production management and decision-making depend heavily on them. Plant disease identification has become a critical issue in recent years. Usually, leaves, stems, flowers, or fruits of disease-infected plants have visible scars or markings. Every disease or pest issue typically has a distinct visual pattern that can be utilized to identify anomalies. Most disease symptoms can initially manifest on the leaves of plants, making the leaves the primary source of information when diagnosing plant disease (Li et al., 2021). **Figure 1** shows the different steps involved in intelligent agriculture systems in smart cities to detect plant diseases.

**Figure 2:**
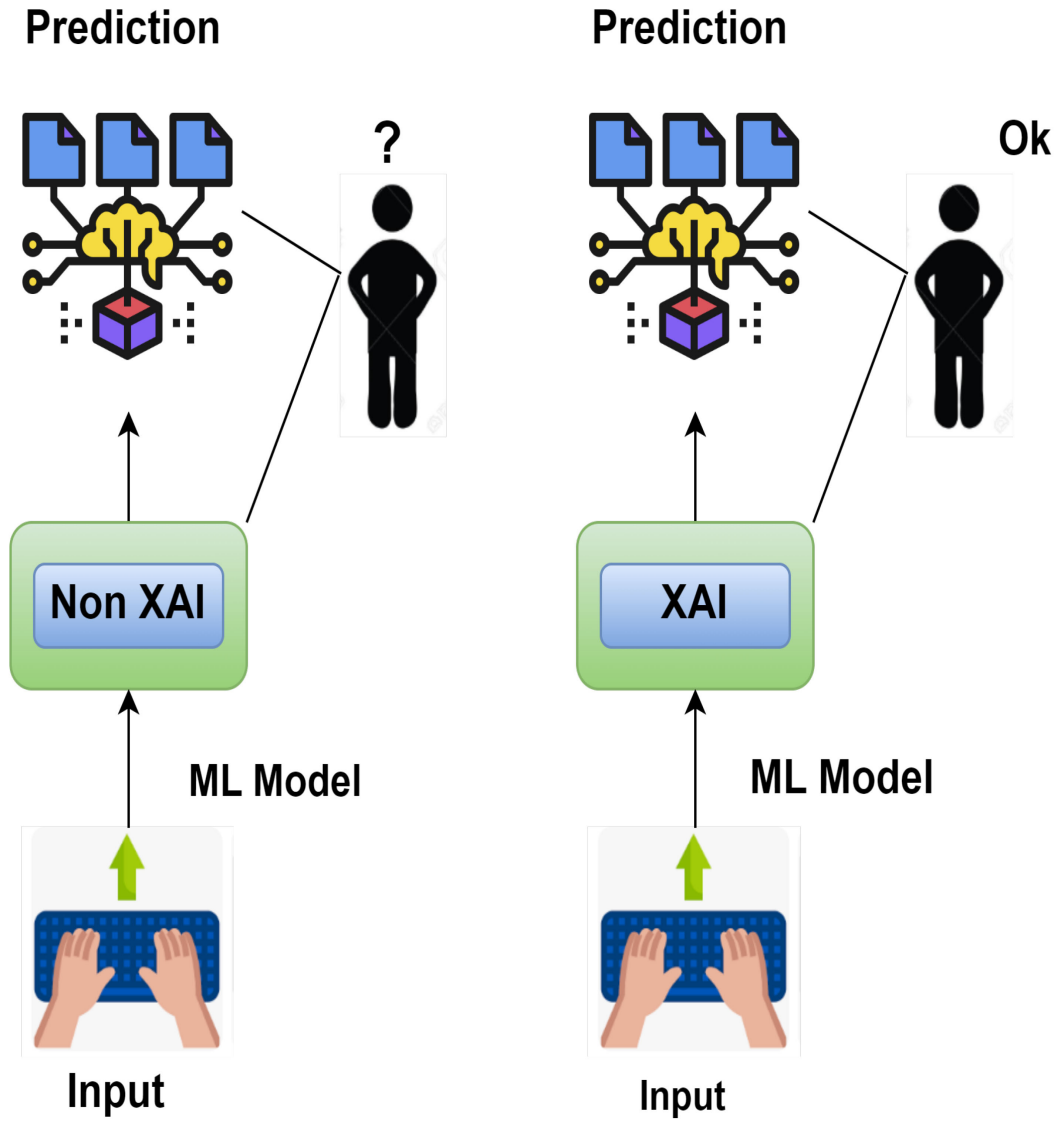
Comparison between XAI and non-XAI.

Smart agriculture utilizes various sensors to collect data (Rajamohana et al., 2024) that can be used to make better decisions and increase crop production. This generates large datasets that can be processed and analyzed by artificial intelligence (AI) and machine learning (ML) algorithms with high accuracy. While developing these algorithms improves decision-making, it will take more time to fully understand and leverage their capabilities. This decision-making process must be transparent so that people can trust AI as a part of their daily routine (Hagras, 2018). Machine learning and interoperability mean presenting machine learning models in a way understandable to humans (Linardatos et al., 2020); Bridgelall, 2024). While interpretability ensures the model is transparent before deployment, explainability explains the black box model *post hoc*. While the definition of interpretability differs from explainability in machine learning, both terms denote more or less the same meaning (Carvalho et al., 2019). **Figure 2** demonstrates the difference between explainable artificial intelligence (XAI) and non-explainable artificial intelligence (non-XAI) by enhancing people’s abilities with broader contextual knowledge, logical inference, and problem-solving, ultimately improving human–machine collaboration (Alsaleh et al., 2023). Systems with non-XAI may find it challenging to convey higher contextual concreteness and transparency, thus limiting their ability to interact and collaborate with humans as shown in **Table 1**.

**Table 1:** Comparison between XAI and non-XAI.

Aspect	Non-XAI	XAI
**Transparency**	Operates as black boxes	Designed for transparency
**Interpretability**	Lack of interpretability	Prioritizes interpretability
**Accountability**	Challenges in accountability	Enhances accountability

XAI is crucial for anticipating corn leaf disease since it gives farmers explicit knowledge of the logic of the predictions. This transparency helps farmers manage their crops.

To effectively safeguard their crops, it is essential to enhance farmers' confidence in AI systems. So, XAI facilitates knowledge sharing and collaboration among academics, agronomists, and farmers, which support the development of more effective disease prediction models and farming methods tailored to corn leaf production.

This study focuses on leveraging explainable AI for diagnosing corn leaf diseases, emphasizing early detection, precise diagnosis, and informed decision-making. This technology aims to help farmers improve crop health, minimize yield losses, and optimize resource management in agriculture.

The paper is structured as follows: Section 2 discusses the previous research on corn leaf diseases, disease prediction methods, and the application of AI in agriculture. This section also highlights the limitations of traditional disease prediction approaches. Section 3 provides a detailed description of the methodology employed in this study, including the data collection process, preprocessing techniques, feature selection methods, and model development. It emphasizes using XAI techniques, such as interpretable machine learning algorithms, to predict corn leaf diseases while providing transparent insights into decision-making. Section 4 discusses the study’s findings, including the performance metrics of the XAI model in predicting corn leaf diseases. The results are presented clearly and concisely to understand the extent to which the developed model can predict corn leaf diseases accurately. The conclusion summarizes the study’s key findings and highlights the significance of the research in the context of agriculture and AI.

**Main contributions**

The following are the main contributions of this article:

• This study employed Explainable AI (XAI) to elucidate the decision-making process, setting it apart from previously published works that lacked such transparency.
• This paper effectively presents a visual geometry group 16 (VGG16) model for utilizing a dataset containing images that address four classes of corn leaf diseases: healthy, common rust, blight, and gray leaf spot.
• The incorporation of layer-wise relevance propagation (LRP) enhances the accuracy of the analysis by providing valuable insights into the model’s decision-making process.
• The combination of VGG16 and LRP offers a viable method for perceiving and investigating corn leaf diseases, enabling precise disease classification and facilitating a deeper understanding of the underlying mechanisms influencing the model’s predictions.

## 2 Literature review

In recent times, smart agriculture has been a field of active research. However, it is essential to note that authors have been unable to find a solution with the necessary features of customizability, interpretability, and anomaly detection in the smart agriculture field. In this section, we will discuss the existing literature related to different modules.

Using a convolutional neural network (CNN) model, Yang et al. (2019) assessed maize seedlings by analyzing spectral characteristics in the visible near-infrared region. Each maize variety’s 3,600-pixel samples were utilized for CNN modeling, and an extra 400 samples were used for testing to achieve a correlation coefficient of 0.8219 with chemical methods for cold damage detection.

Agarwal et al. (2019) used a CNN with three convolution layers, three max-pooling layers, and two fully connected layers. The dataset contains corn leaves with three diseases: corn gray leaf spot, corn common rust, and corn northern leaf blight, and obtained an accuracy of 94%.

Zhen et al. (2020) used regression-guided detection network (RDNet) with the VGG16 model as a foundation and replaced the global pooling layer with a fully connected layer. Based on the encoder–decoder structure, a regional segmentation network (RSNet) was created. The use of multi-scale kernels of varying sizes enabled the model to detect different features on different scales. The shallow field of the original convolution kernel is near the given image and accurately isolates the suspect area. Segmentation experiments were conducted on a dataset comprising field photographs of various crop diseases such as corn leaf spot, corn round spot, wheat stripe rust, wheat anthracnose, cucumber target spot disease, and cucumber anthracnose. This model achieved an accuracy of 87.04%.

Saeed et al. (2021) proposed an automated crop disease detection system by using partial least squares (PLS) for the feature selection. The authors used the pre-trained VGG19 network to extract-deep features from the plant village dataset, which included images of tomato, corn, and potato. Then, the PLS parallel fusion approach was employed to merge the features acquired through the 6th and 7th layers of the VGG19 network. Moreover, the PLS method was used to select the best features and achieved an accuracy of 90.1%.

A study by Sandotra et al. (2023) included the implementation of pre-trained DL models for corn leaf disease detection and compared various CNN architectures. Residual network (ResNet50), VGG16, VGG19, InceptionV3, and EfficientNetB0 were trained and used on a leaf dataset of corn leaf, and achieved accuracy rates of 70.02%, 91.37%, 89.69%, 87.77%, and 92.33%, respectively.

**Table 2** shows the different AI models for the diagnosis of corn leaf disease, involving their types, accuracies, limitations, and their applied datasets. Two authors used the CNN model that was applied to two different studies based on supervised learning, and reached accuracy rates of 82.19% and 94% on the hyperspectral data and the multi-corn leaf diseases, respectively. Other studies employed the RDNet method using unsupervised learning, achieving an accuracy of 84.04% in detecting both corn leaf spot and corn round spot. Some authors focused on the fusion of VGG19, CNN, and PLS using supervised learning. It gave an accuracy of 90.01% on the images of tomato, corn, and potato. The last one used five different CNN models to detect corn leaf disease. So, all the given models did not use XAI to predict corn leaf disease.

**Table 2:** Comparison of different AI models used to predict corn leaf disease.

Ref.	Model	Model type	Accuracy (%)	Applied on	Limitations
Yang et al. (2019)	CNN	Supervised learning	82.19	hyperspectral images	Non-Explainable Artificial Intelligence (XAI) Used
Agarwal et al. (2019)	CNN	Supervised learning	94	corn gray leaf spot, corn common rust, corn northern leaf blight, and healthy	
Zhen et al. (2020)	RDNet	Un- supervised learning	84.04	corn leaf spot and corn round spot	
Saeed et al. (2021)	VGG19, CNN, and PLS	Supervised learning	90.01	images of tomato, corn, and potato	
Sandotra et al. (2023)	ResNet50, VGG16, VGG19, InceptionV3, and EfficientNetB0	Supervised learning	70.02, 91.37, 89.69, 87.77, and 92.33	corn blight, corn common rust, corn gray leaf spot, and corn healthy	

## 3 Materials and methods

**Figure 3:**
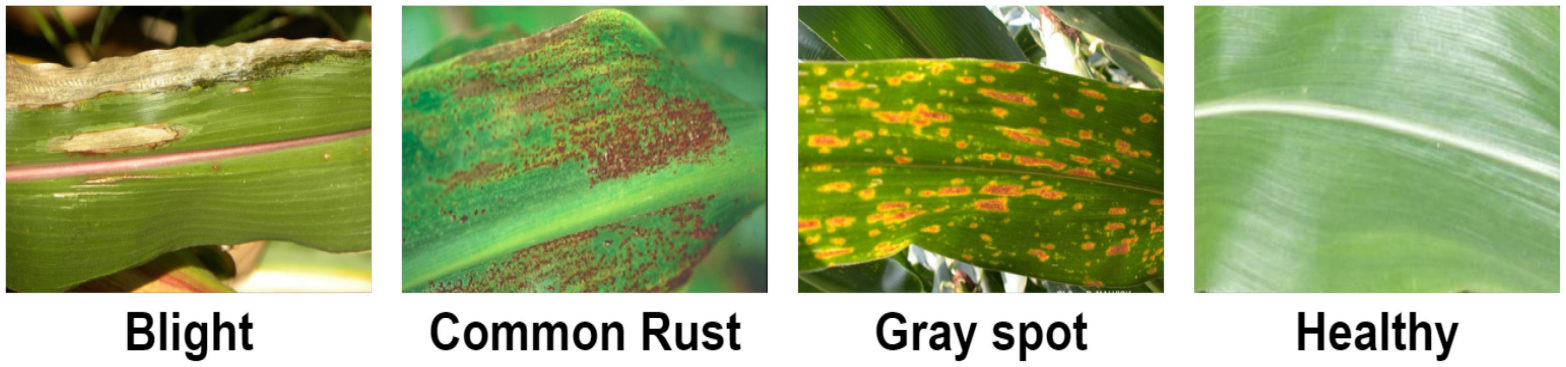
Samples of corn leaf disease (Smaranjit Ghose, 2024).

The dataset contained 4,188 total images that included four corn leaf disease classes. There were 1,146 images of blight leaf, 1,306 images of common rust, 574 images of gray spot, and 1,162 images of healthy leaf, and this dataset was divided randomly into 70% (2,930 images) for training and 30% (1,258 images) for testing. The data were acquired from the Kaggle repository (Smaranjit Ghose, 2024). **Figure 3** shows the samples of corn leaf disease.

**Figure 4:**
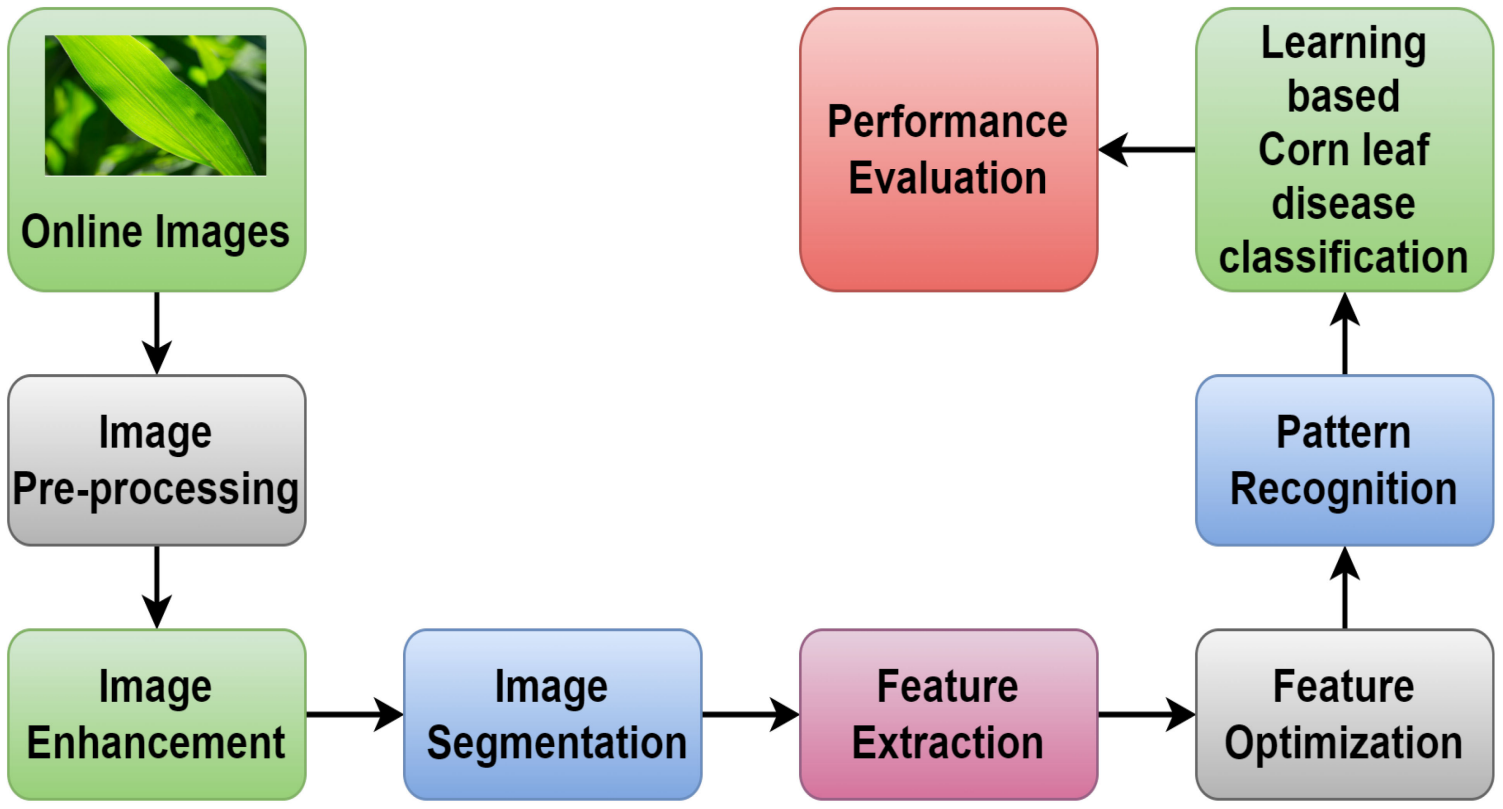
A general view of the prediction flow.

Image classification involves several steps: First, a labeled dataset of images is created. Second, the images are preprocessed by way of resizing, normalizing, and augmenting them; then, the features are extracted using pre-trained CNN or other methods. On the basis of the extracted features and their corresponding labels, a model is trained, after which it is validated for generalization, fine-tuned as required, and evaluated on a different test dataset. Lastly, the trained model is put to use in the real world, which classifies the new images by their visual content. This iterative process enables the creation of robust models for tasks such as object detection, classification, segmentation, and generation across diverse domains (Fadhilla et al., 2023). **Figure 4** is an illustrated representation of this process.

**Figure 5:**
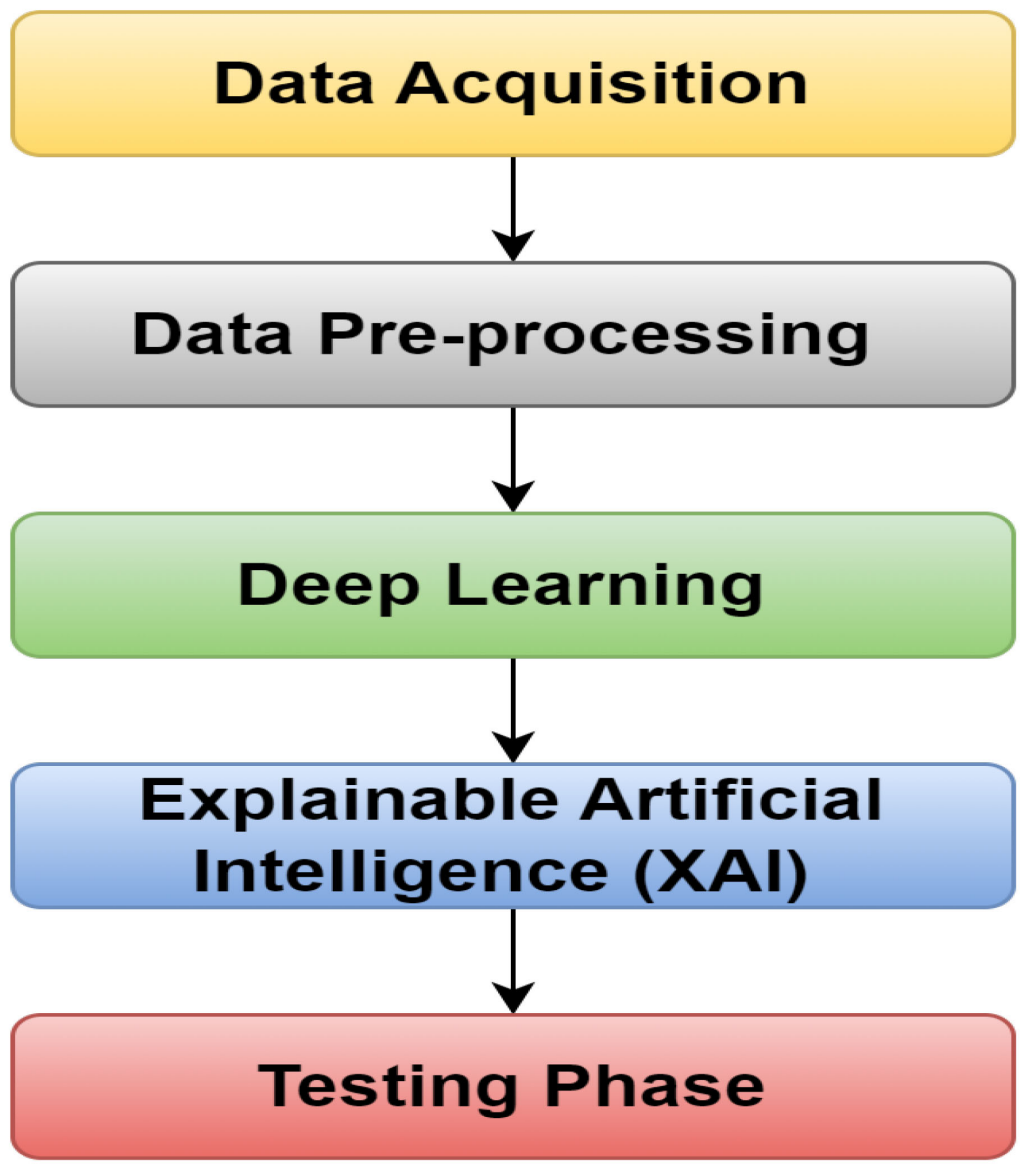
Five steps of the proposed model.

The proposed model design has two phases: training testing. This design contains five essential steps, as shown in **Figure 5**. In the first step, the data are incorporated, and then used for preprocessing in the second step. After applying DL models to the data, the XAI model is used to explain the results. Ultimately, the last step ensures the model’s performance.

**Figure 6:**
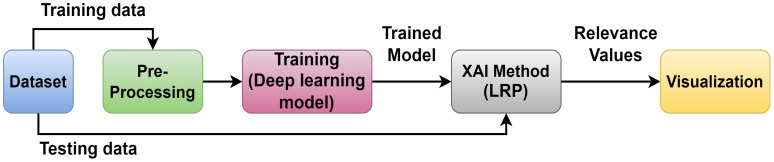
A general view of the proposed model.

**Figure 6** depicts the architecture of the proposed approach, providing a general overview. The dataset (Smaranjit Ghose, 2024) is processed in two phases: training and testing. So, a DL model is used for training in which the VGG16 model is employed; after that, XAI techniques are implemented to visualize the essential features using the trained model employing LRP. This study used the LRP method for XAI. LRP, one of the primary algorithms for network explainability, uses the backpropagation algorithm (Bach et al., 2015). LRP explains a classifier’s prediction for a specific data point by attributing ‘Relevance values’ (Ri) to important input components using the topology of the trained model. It is effective for images, videos, and text (Ullah et al., 2021). The DL model is used for the basic prediction of preprocessed data (Makridakis et al., 2023). The XAI model contrasts these expectations and the preprocessed data and utilizations for the correlations to make sense of the prediction made by the DL model. So, the clarifications given by the XAI model are good and show fair thinking behind the prediction, and the testing data are applied to the trained model to check the performance of the model.

**Figure 7:**
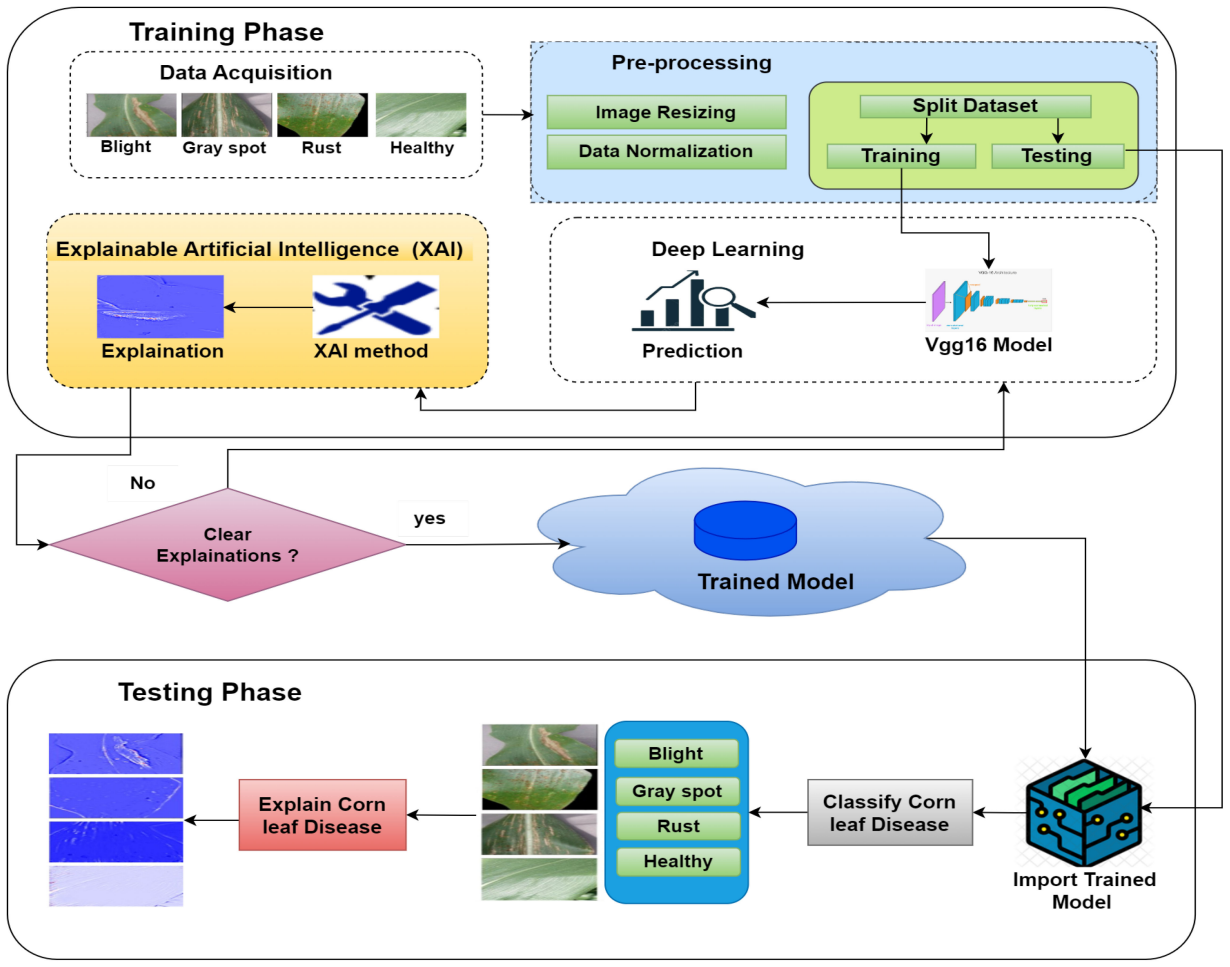
Detailed proposed model for predicting corn leaf disease with XAI using VGG16.

The proposed model for detecting corn leaf disease, which incorporates XAI, is presented in detail in **Figure 7**. In this proposed model, during the training phase, the data acquisition step is responsible for obtaining the raw dataset (Smaranjit Ghose, 2024) of corn leaf disease images from the Kaggle repository. This dataset includes four categories: blight, gray spot, common rust, and healthy. The initial step is to preprocess the raw dataset. This involves resizing the images and normalizing the data. After that, the dataset is then divided into training and testing steps by the requirements of the DL model implementation. In this approach, the CNN–VGG16 model used for this research includes a convolution layer, a pooling layer, a dropout layer, a flattened layer, and a dense layer (Asriny and Jayadi, 2023); Mardiana et al., 2023). The VGG16 model was modified to include the four classes required by the sample dataset (Smaranjit Ghose, 2024). During the testing phase, the testing dataset is used for assessment. The trained model stored in the cloud is then used to classify the corn leaf diseases into four classes: blight, gray spot, common rust, and healthy, which explain the corn leaf disease.

In this study, a previously trained VGG16 model was used for transfer learning in AI. This technique is precious for AI developers as it gives them a shortcut to building good models, which is a bonus for both time and computer resources. The process usually involves the utilization of the VGG16 as a feature extractor, which in turn captures the particular characteristics of images related to the new task from the dataset. This model has several advantages, such as better performance, a smaller amount of labeled data, and efficient use of computational resources. So, transfer learning with pre-trained models such as VGG16 basically makes the development of AI applications easier by using previous knowledge for adaptation to the new tasks (Özden, 2023).

XAI in predictive modeling is the key to the enhancement of the transparency and the trust of the users by giving them information about the decision-making process of an AI model. XAI methods are designed to make complicated models comprehensible to people, thus, it would be easy for users to understand why a particular prediction is made. These techniques produce explanations by focusing on the vital attributes, visualizing the model behavior, or providing context-oriented insights specific to the application domain. Through the introduction of XAI into predictive models, stakeholders will be able to not only acquire useful information about model predictions but also reduce the risks related to bias, mistakes, and the lack of transparency, hence fostering the acceptance and trust of AI systems in the real world (Ullah et al., 2021).

As shown in **Table 3**, the pseudo-code frames a proposed model for foreseeing corn leaf diseases utilizing XAI with the VGG16 calculation. The interaction includes two phases: training and testing. In the training phase, the dataset (Smaranjit Ghose, 2024) is gathered from Kaggle and then split into training and testing sets. A DL model is then applied to the training dataset, and the model’s forecasts are done by utilizing XAI procedures. If the clarifications meet the standards for agreeable execution, the trained model is stored in the cloud. If not, it sets off a retraining cycle. In the testing phase, testing data are used with the trained model and then predict the corn leaf disease. After that, the reasons for corn leaf disease are explained, and such are quite helpful for farmers in smart agriculture systems.

**Table 3:** The pseudo-code of the proposed model with XAI using VGG16.

Training phase
1-Data Acquisition *raw_dataset* = *acquire_raw_dataset_from_Kaggle*() 2-Preprocessing preprocessed_dataset = *preprocess_dataset*(*raw_dataset*) Splitting into training and testing sets *training_set, testing_set* = *split_dataset*(*preprocessed_dataset*) 3-Deep Learning *trained_model* = *train_deep_learning_model*(*training_set*) 4- XAI explanations = explain_predictions(trained_model, testing_set) **If** *explanations_are_satisfactory(explanations)* Store_model_in_cloud(trained_model) ** Else** retrain_model() ** EndIf**
Testing phase
5-Testing raw_testing_data = collect_raw_testing_data() preprocessed_testing_data = preprocess_testing_data(raw_testing_data) Classification using the trained Model classified_data = classify_data_using_trained_model(trained_model, preprocessed_testing_data) Import and utilize identified and predicted data import_and_utilize_data(classified_data)

## 4 Simulation and results

Some key metrics were evaluated to critically examine various aspects of the model’s performance. These include accuracy, precision, true positive rate, false positive rate, and misclassification rate (Shahinfar et al., 2020). Accuracy is a performance metric that measures how well a model classifies images, regardless of the classification error type (Valero-Carreras et al., 2023).


(1) $$Accuracy=\frac{TP+TN}{TP+TN+FP+FN}*100$$

TP, FP, TN, and FN are symbols that indicate true positives, false positives, true negatives, and false negatives, respectively (Heydarian et al., 2022).

Precision measures how many images were correctly classified by the model as a fraction of the total number of images classified (Heydarian et al., 2022).


(2) $$Precision=\frac{TP}{TP+FP}*100$$

The false negative rate measures the fraction of incorrectly classified images from all negative pictures (Renshaw, 1997).


(3) $$False\_negative\_rate=\frac{FN}{FN+TP}*100$$

In a confusion matrix, specificity is calculated by taking the TN for a given class and dividing it by the sum of TN and FP for that class (Van Stralen et al., 2009).


(4) $$Specificity=\frac{TN}{TN+FP}*100$$

The misclassification rate in the confusion matrix represents the proportion of cases classified incorrectly by the model (Llullaku et al., 2009).


(5) $$Misclassification\_rate=\frac{FN+FP}{TP+TN+FP+FN}*100$$

The evaluation metrics are used to measure the efficiency and effectiveness of the proposed approach, as shown in Equations 1–5.

The suggested model classifies images of corn leaf diseases into four categories: blight, common rust, gray leaf spot, and healthy. The model’s training parameters are the number of epochs, optimization algorithm, input image size, batch size, and learning rate, as shown in **Table 4**.

**Table 4:** Training parameters.

Training parameters	Values
**No. of epochs**	10
**Batch size**	32
**Learning rate**	0.0001
**Optimization algorithm**	Adam
**Input image size**	224 × 224 × 3

**Figure 8:**
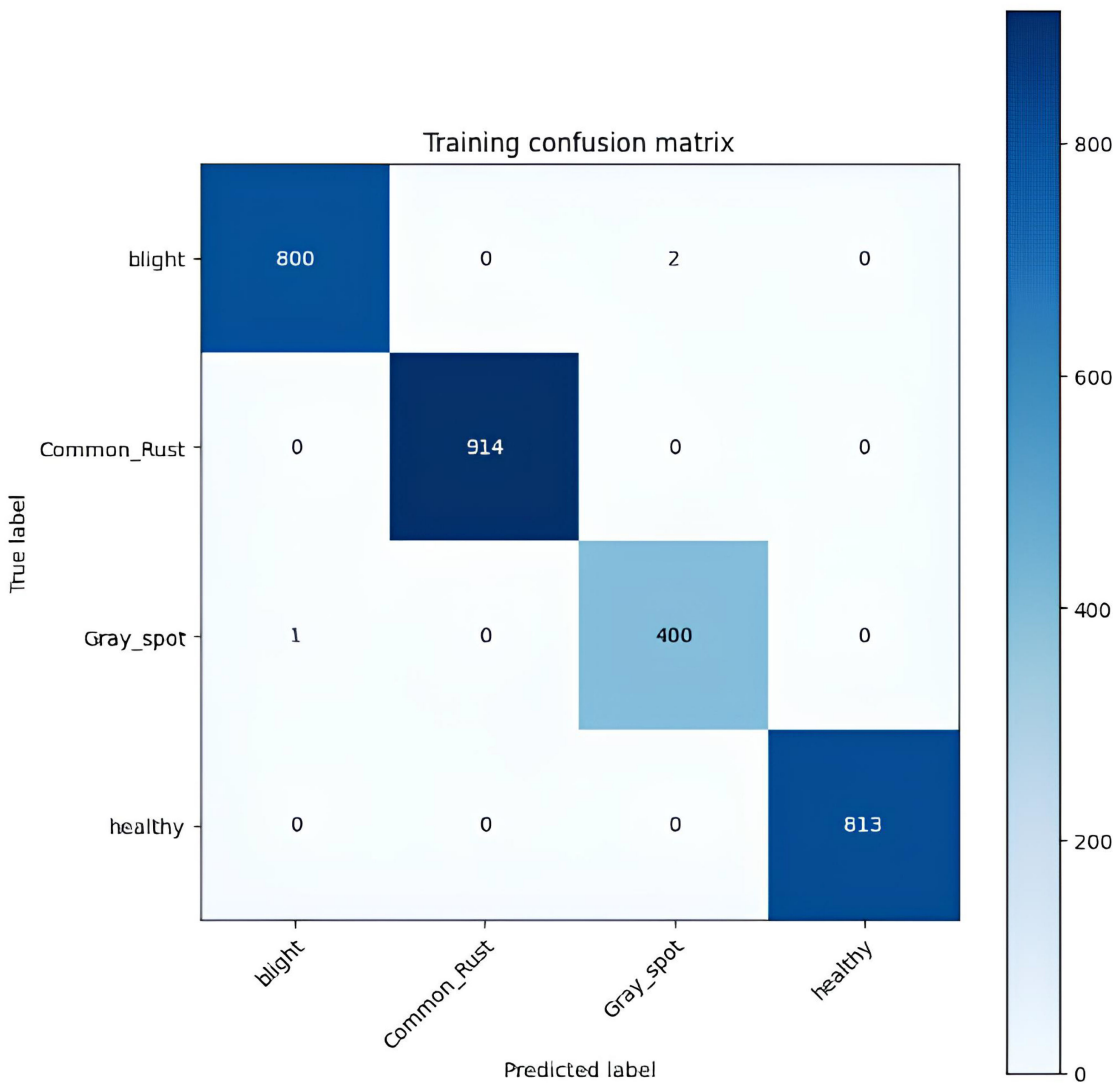
Training confusion matrix of the proposed model.

**Table 5** shows a confusion matrix generated during the training process for classifying corn leaf diseases. The matrix lists the actual classes as rows and the predicted classes as columns. Each matrix cell represents the number of instances classified accordingly during training. For example, the model accurately classified 800 instances of blight, 914 instances of common rust, 400 cases of gray leaf spot, and 813 instances of healthy. The off-diagonal elements of the matrix indicate the misclassifications, such as two instances of blight being incorrectly classified as gray leaf spot and one example of gray leaf spot being misclassified as blight. This matrix is a valuable tool for evaluating the performance of the classification model, helping to identify areas of accurate classification and pointing where errors occur, so the training results of the model are as illustrated in **Figure 8**.

**Table 5:** Training confusion matrix of the proposed model.

Actual/predicted	Blight	Common rust	Gray leaf spot	Healthy
**Blight**	800	0	2	0
**Common rust**	0	914	0	0
**Gray leaf spot**	1	0	400	0
**Healthy**	0	0	0	813
**Training accuracy**	99.89%			

**Figure 9:**
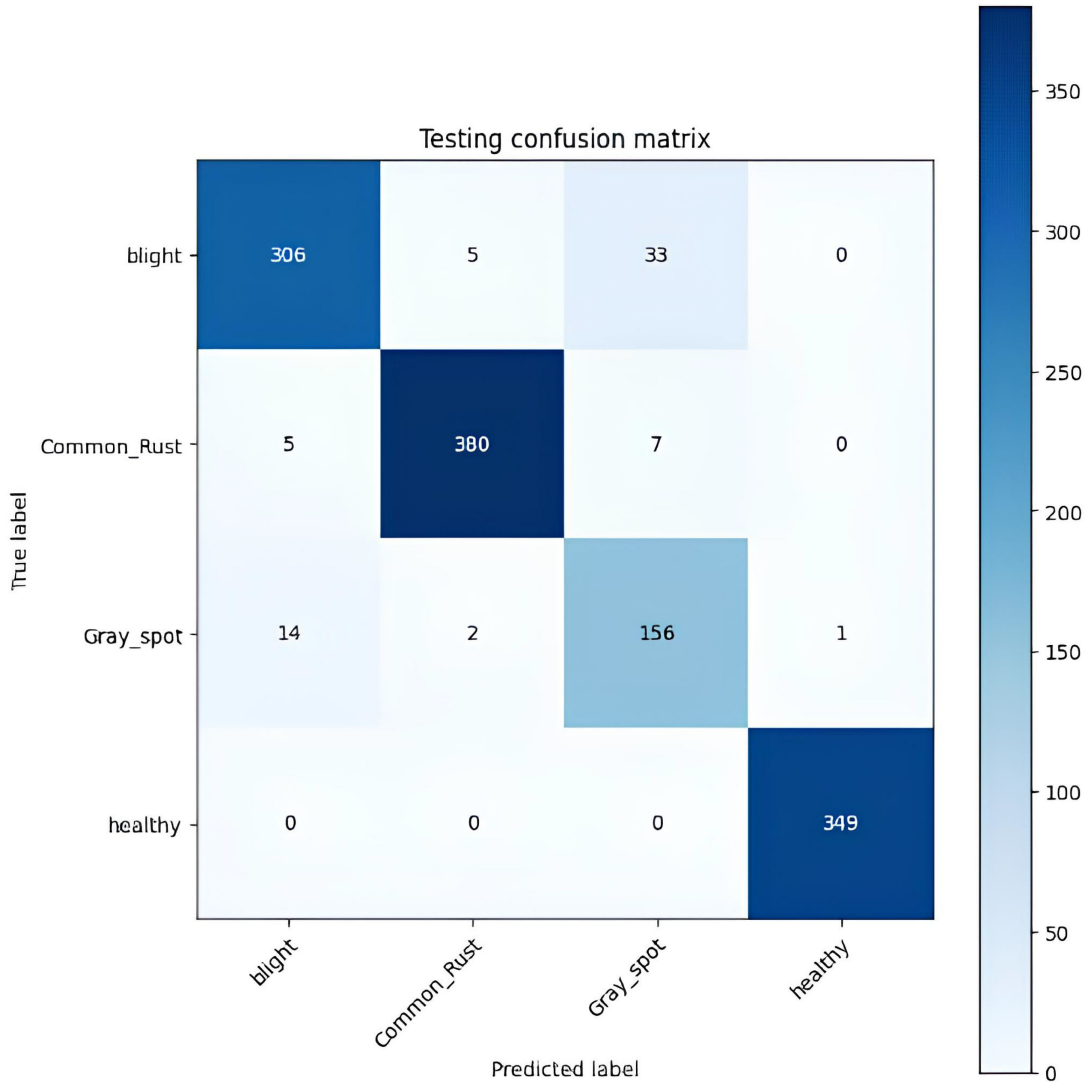
Testing confusion matrix of the proposed model.

**Table 6** displays the confusion matrix for the corn leaf disease classification test. The rows represent the actual classes, whereas the columns indicate the predicted classes. Each cell in the table displays the count of instances classified accordingly during the testing phase. For example, the model correctly classified 306 instances of blight, 380 cases of common rust, 156 cases of gray leaf spot, and 349 instances of healthy. However, some misclassifications were observed, such as 33 instances of gray leaf spot being misclassified as blight and seven common rust misclassified as gray leaf spot. The testing accuracy, shown at the bottom of the table, is 94.67%. This represents the proportion of correctly classified instances from the total testing dataset. This matrix provides valuable insights into the model’s performance, identifying areas where misclassifications occur. It helps further refine and evaluate the classification model, as shown in **Figure 9**.

**Table 6:** Testing confusion matrix of the proposed model.

Actual/predicted	Blight	Common rust	Gray leaf spot	Healthy
**Blight**	306	5	33	0
**Common rust**	5	380	7	0
**Gray leaf spot**	14	2	156	1
**Healthy**	0	0	0	349
**Testing accuracy**	94.67%			

**Table 7** presents the per-class performance metrics of a classification model used to identify four different classes of corn leaf disease. The performance metrics, namely, accuracy, precision, false negative rate, specificity, and misclassification rate are all expressed as percentages. The model shows high performance over all the classes in the training phase. In the blight class, accuracy is 99.98%, precision is 99.87%, and specificity is 99.95%, where the false negative rate is 0.24% and misclassification rate is 0.1023%; common rust and healthy class show 100% accuracy, precision, and specificity, and have 0% false negative rate and misclassification rate; and in the gray leaf spot, the accuracy is 99.89%, precision is 99.50%, and specificity is 99.92%, where the false negative rate is 0.249% and misclassification rate is 0.1023%. Blight and gray leaf spots show equal accuracy and misclassification rates. Overall, the model shows high accuracy in identifying corn leaf diseases in the training phase.

**Table 7:** Per-class performance metrics in training.

Classes	Accuracy (%)	Precision (%)	False negative rate (%)	Specificity (%)	Misclassification rate (%)
**Blight**	99.89	99.87	0.24	99.95	0.1023
**Common rust**	100	100	0	100	0
**Gray leaf spot**	99.89	99.50	0.249	99.92	0.1023
**Healthy**	100	100	0	100	0

**Table 8** provides a detailed evaluation of a classification model’s performance on the four classes of corn leaf disease in the testing phase. The performance of each class is assessed using five key metrics, namely, accuracy, precision, false negative rate, specificity, and misclassification rate. In the blight class, the accuracy is 95.46%, precision and specificity are 94.15%, the false negative rate is 11.046%, and the misclassification rate is 4.53%. The common rust class achieved 98.48% accuracy, precision is 98.19%, and specificity is 99.19%. The gray leaf spot has the same accuracy as the blight class, but the precision is 79.59%, the false negative rate is 9.82%, the specificity is 96.31%, and the misclassification rate is 4.53%. In the healthy class, the accuracy is 99.92%, precision is 99.71%, specificity is 99.88%, false negative rate is 0%, and misclassification rate is 0.07%. The model shows excellent results with respect to classifying different classes of corn leaf disease in the testing phase.

**Table 8:** Per-class performance metrics in testing.

Classes	Accuracy (%)	Precision (%)	False negative rate (%)	Specificity (%)	Misclassification rate (%)
**Blight**	95.46	94.15	11.046	94.15	4.53
**Common rust**	98.48	98.19	3.06	99.19	1.51
**Gray leaf spot**	95.46	79.59	9.82	96.31	4.53
**Healthy**	99.92	99.71	0	99.88	0.07

**Figure 10:**
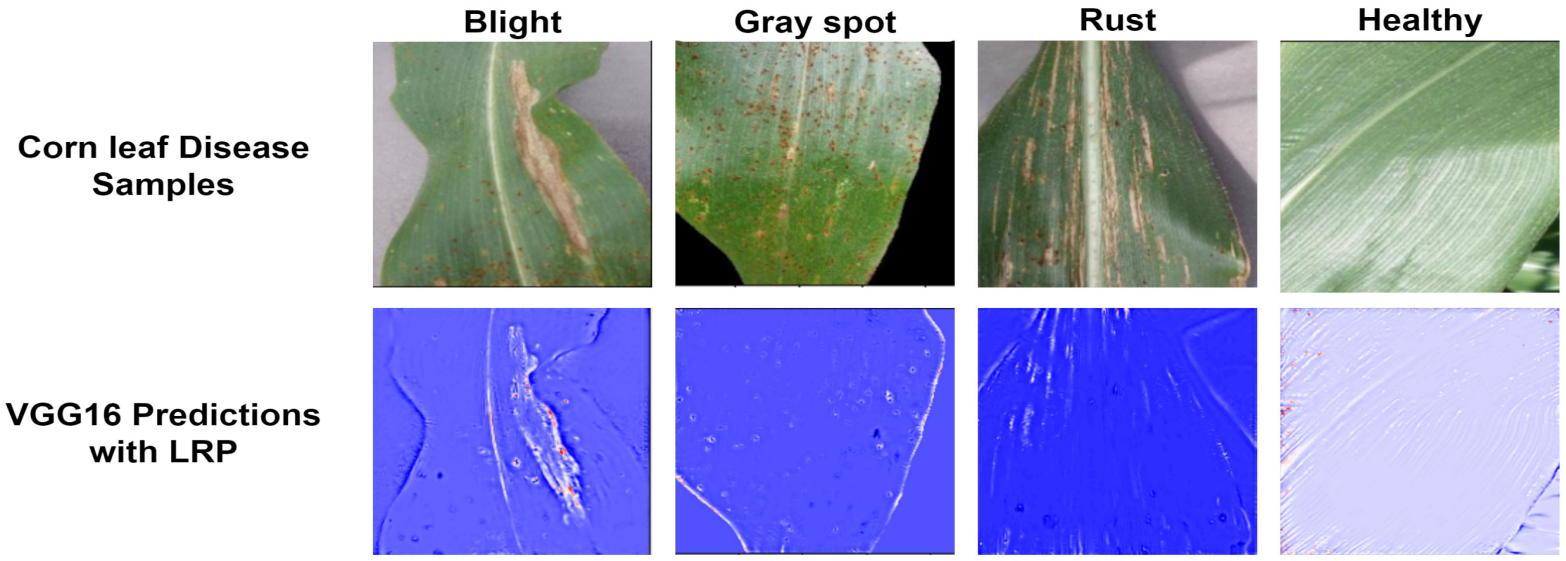
Results of the proposed model.

**Table 9** displays the proposed model’s performance metrics for training and testing. **Figure 10** shows the results of the VGG16 model enhanced with LRP. This technique helps us understand the decisions made by the model. In this study, the LRP and VGG16 models are used to predict different types of corn leaf diseases, such as healthy, blight, common rust, and gray leaf spot. LRP generates results that allow us to understand the features and regions within the corn leaf images that contribute most significantly to the model decision-making process. This approach helps us interpret the model predictions more transparently and explainable, making it possible for researchers and practitioners to test the model performance. It also helps identify areas that require improvement or refinement in the classification task.

**Table 9:** Overall performance metrics of training and testing.

Performance metrics	Training (%)	Testing (%)
**Accuracy**	99.89	94.67
**Precision**	99.84	92.91
**False negative rate**	0.12	5.98
**Specificity**	99.96	98.32
**Misclassification rate**	0.11	5.33

**Table 10** compares the different models used to predict corn leaf disease, showing their accuracy, loss rate, and whether they used XAI. The suggested model used VGG16 with LRP and reached 94.67% accuracy. This is the only model that uses XAI to give transparency in the decision-making process. In other models compared, different AI models had lower accuracies and did not use XAI techniques. So, the proposed model achieves a good balance between high performance and interpretability using XAI.

**Table 10:** Comparison of the proposed model with related works to predict corn leaf disease.

Ref.	Model	Accuracy (%)	Misclassification rate (%)	XAI
Yang et al. (2019)	CNN	82.19	17.81	Non-Explainable Artificial Intelligence (XAI) Used
Agarwal et al. (2019)	CNN	94	6	
Zhen et al. (2020)	RDNet	84.04	15.96	
Saeed et al. (2021)	VGG19, CNN, And PLS	90.01	9.99	
Sandotra et al. (2023)	ResNet50, VGG16, VGG19, InceptionV3, and EfficientNetB0	70.02, 91.37, 89.69, 87.77, and 92.33	29.98, 8.63, 10.31, 12.23, and 7.67	
**Proposed** **model**	**VGG16 empowered with** **LRP**	**94.67**	**5.33**	**Yes**

Bold values show the results of the proposed model.

## 5 Conclusion

In this paper, the model VGG16 is employed to deal with the images used to detect the disease of corn leaves. This model achieves a better performance in terms of accuracy, specificity, misclassification rate, and false positive rate with respect to previously published works. The LRP with VGG16 model is used to accurately diagnose corn leaf diseases in agriculture. This technique makes it possible for farmers to get information regarding what is happening with corn leaf diseases at the current moment. This model enables farmers to take action at the right time and utilize various resources efficiently. This method to detect disease in the early steps can be used to prevent crop damage. Therefore, this model enhances farmers’ understanding of disease transmission or crop management and other facets of sustainable agriculture with the help of proper explanations and visualizations. This study demonstrates the relevance of XAI in smart agriculture and acts as a foundation for future studies on how explainable methods can be employed to achieve further improvements in the performance and reliability of deep neural networks in agriculture.

## Data availability statement

The raw data supporting the conclusions of this article will be made available by the authors, without undue reservation.

## Author contributions

MT: Conceptualization, Software, Writing – original draft. UA: Formal analysis, Software, Visualization, Writing – review & editing. SA: Formal analysis, Methodology, Supervision, Writing – original draft. SH: Data curation, Formal analysis, Software, Writing – review & editing. RN: Funding acquisition, Software, Visualization, Writing – review & editing. MK: Investigation, Methodology, Project administration, Supervision, Writing – original draft. DJ: Data curation, Funding acquisition, Resources, Software, Writing – review & editing.
